# Defending Others Online: The Influence of Observing Formal and Informal Social Control on One’s Willingness to Defend Cyberhate Victims

**DOI:** 10.3390/ijerph20156506

**Published:** 2023-08-02

**Authors:** Matthew Costello, James Hawdon, Ashley V. Reichelmann, Atte Oksanen, Catherine Blaya, Vicente J. Llorent, Pekka Räsänen, Izabela Zych

**Affiliations:** 1 Department of Sociology, Anthropology & Criminal Justice, College of Behavioral, Social and Health Sciences, Clemson University, Clemson, SC 29634, USA; 2 Center for Peace Studies and Violence Studies, Department of Sociology, College of Liberal Arts and Human Sciences, Virginia Tech, Blacksburg, VA 24061, USA; hawdonj@vt.edu (J.H.); avr@vt.edu (A.V.R.); 3 Faulty of Social Sciences, Tampere University, 33100 Tampere, Finland; atte.oksanen@tuni.fi; 4 URMIS, Department of Education Sciences, Université Côte d’Azur, 06103 Nice, France; catherine.blaya@univ-cotedazur.fr; 5 Department de Educación, University of Cordoba, 14001 Cordoba, Spain; vjllorent@uco.es (V.J.L.); izych@uco.es (I.Z.); 6 School of Economics, University of Turku, 20100 Turku, Finland

**Keywords:** cyberhate, social control, self-help, online hate

## Abstract

This paper examines factors correlated with online self-help—an informal form of social control vis-à-vis intervention—upon witnessing a cyberhate attack. Using online surveys from 18- to 26-year-old respondents in the United States, we explore the roles of various types of online and offline formal and informal social control mechanisms on the enactment of self-help through the use of descriptive statistics and ordinal logistic regression. The results of the multivariate analyses indicate that online collective efficacy is positively related to self-help, as is having close ties to individuals and groups offline and online. Formal online social control, however, is not significantly related to engaging in self-help. Other findings demonstrate that personal encounters with cyberhate affect the likelihood that an individual will intervene when witnessing an attack, and that individuals with high levels of empathy are more likely to intervene to assist others. This work indicates that pro-social online behavior is contagious and can potentially foster online spaces in which harmful behaviors, such as propagating cyberhate, are not condoned.

## 1. Introduction

Evolving information and communication technologies continue to redefine the parameters of human relationships, easing the constraints of physical distance and allowing for theoretically limitless engagement [1]. Unfettered virtual interaction creates new risks, however, including those associated with the rising tide of abusive online behavior [2,3,4]. Indeed, a recent survey found sixty-seven percent of young people and forty-nine percent of older adults have been targets of cyber-harassment [5]. These figures are concerning because victims of cyber-abuse can suffer myriad harms, ranging from depression, fear and low self-esteem to self-harm and outwardly directed violence [6,7,8,9,10,11,12]. It is therefore vital to explore effective techniques to both reduce the likelihood of harmful online interactions and mitigate their impact on victims. To that end, researchers are increasingly interested in the role of online bystanders in confronting cyber-abuse. Given their proximity to cyber-attacks, online bystanders are often the first line of potential defense, and their response—or lack thereof—can affect the severity and duration of an attack.

The circumstances under which individuals intercede to aid individual in distress have been rigorously explored in offline settings (for a comprehensive overview, see [13,14]). However, the nature and efficacy of bystander intervention online is less well understood, and much of what is known is grounded in work conducted in offline settings [15,16,17,18,19] and disproportionately focuses on bystander responses by adolescents to select forms of cyber-abuse [20,21,22,23,24,25]. The current study contributes to the extant literature on online bystander intervention by examining self-help in young adults who encounter online hate material, or cyberhate. Self-help is a specific form of informal social control whereby individuals handle a grievance with unilateral aggression [26], and we are particularly interested in how various forms of formal and informal social control can encourage or discourage respondent self-help via online bystander intervention. The dynamic between formal and informal social control in relation to online self-help has not been adequately explored, and we therefore believe this study offers a novel contribution to the pertinent literature. In particular, we assess if witnessing either formal or informal social control online affects the likelihood that an online user will likewise try to intercede to assist victims. To explore this issue, we draw on the current understanding of conditions that relate to bystander intervention, while also exploring the possibility that formal and informal social control may be inversely related to one another. Understanding the circumstances under which self-help is enacted is critical as both Internet usage and harmful online material are on the rise. Indeed, developing knowledge on what leads to self-help is a critical first step in trying to reduce harmful online content and creating civil online spaces.

### 1.1. Bystander Intervention

Most bystanders who witness others in need decide not to offer assistance. Researchers have outlined a number of situational and personal factors to help to explain why. Importantly, the presence of other bystanders adversely affects the probability of intervention by increasing anonymity and diffusing the responsibility of each individual to act [13,27,28]. Fear that others will judge one’s actions negatively also impedes intervention, particularly in large crowds with more potential evaluators [29,30]. Additionally, bystanders can take cues from other bystanders, and the inaction of others may signal that assistance is not necessary, leading to a state of pluralistic ignorance rooted in cascading faulty assumptions [18,31,32]. Other considerations, including incident severity [33], a bystander’s age [34], gender [35], education [36], religiosity [37], diffidence [30], empathy [38], history of victimization [34], social support system [39], and connection to the victim [36], can also affect the likelihood of intervention.

Bystander behavior has been examined across numerous experimental and real-world settings involving various medical emergencies, thefts, smoke-filled rooms, car crashes, graffiti, interpersonal violence, bullying, and fainting (for a comprehensive overview, see [13,14]). Yet, the Internet, especially social media, raises critical new questions about the conditions under which bystanders intervene to assist fellow online users in need as well as the outcomes of such interventions. Existing work on online bystander intervention suggests that, indeed, online helping behavior is inhibited by the presence of others in virtual spaces [24,40]. Personal and situational factors are likewise shown to influence online bystander behavior. Online bystanders are more likely to intervene when cyber-attacks are deemed more serious, they feel connected to the victim [20,33,41], and victims ask for help [42]. Females have been found to be more likely to intervene online to help others, as are those with higher levels of self-control and empathy, individuals with strong familial bonds, prior online victims, witnesses to pro-social online behavior [41,43,44], and individuals exposed to bystander intervention programs [45].

The current study offers a novel contribution to the extant work on online bystander behavior by exploring a unique form of cyberviolence, cyberhate, and spotlighting the role of several social control mechanisms on online bystander intervention. Cyberhate targets individuals in the aggregate, distinguishing it from other forms of problematic online behavior, such as cyberbullying, cyberstalking, or cyberharassment, which target specific individuals for idiosyncratic reasons. Victims of cyberhate are targeted based on group characteristics, including race, immigrant status, religion, gender, and sexuality [46]. Exploring bystander interventions is especially important when cyberhate is at issue because most hateful online content is protected freedom of speech in the United States and the government, therefore, plays a minimal role in its regulation [47,48]. Moreover, efforts by social media companies to control hateful content on their platforms are often inadequate, while some networking sites eschew efforts to police content altogether. Hence, the burden of regulating online spaces typically falls on users [49].

### 1.2. Formal and Informal Social Control

Targeting outgroup members [50], cyberhate can erode trust between communities and undercut social cohesion [51]. Yet, it is possible that responses to cyberhate can actually build trust between individuals and discourage further abusive online behavior. We explore this possibility by examining whether social control mechanisms encourage pro-social behavior intended to combat cyberhate. Self-help is a type of informal social control whereby individuals handle a grievance with unilateral aggression [26]. It can range from a simple statement of disapproval to the use of violence to impose one’s will. We are interested in understanding why some online users engage in self-help when they encounter cyberhate, especially since doing so involves risks. Not only can intervening to assist a victim be unsuccessful, leading to lost time and wasted effort, but it can also generate embarrassment on the part of the intervener or disapproval from other online bystanders [52,53,54]. Additionally, enacting self-help can exacerbate the abusive behavior the intervener is trying to abate [55] or even render the intervener the target of unwanted behavior [48].

However, while the presence of other bystanders can reduce the likelihood that an observer will intervene in defense of another or might even encourage and contribute to further victimization [20], witnessing others intervene to protect the community can potentially embolden others. In essence, victims with partisans are more likely to be defended [33], fostering collective efficacy. Collective efficacy is informal social control enacted by others and is defined by mutual trust and a shared willingness to intervene for the common good [56,57]. Offline, collective efficacy has demonstrated the ability to reduce crime and delinquency [57,58,59,60], though its utility at deterring harmful behavior online remains unclear. While online collective efficacy can signal that abusive behavior is not acceptable in a virtual space and will be met with shared resistance, fostering collective efficacy in cyberspace faces challenges. This could partly be due to the unknown size of the group, as previous work finds that group size affects the likelihood of intervention [61]. More likely, mutual trust, essential to collective efficacy, is often lacking online. Sampson and colleagues [57] argue that individuals are less apt to intervene on behalf of those who they do not trust, and the anonymity of the Internet, coupled with the sporadic nature of online interactions, makes building trust difficult. Anonymity also lowers the potential costs of engaging in abusive behavior; whereas abusers in the physical world can potentially face shame, embarrassment, or even physical harm for their actions, these consequences are easily eschewed by faceless online users [48]. Despite the difficulties inherent in fostering collective efficacy online, we expect it to have a positive effect on helping behavior when it does manifest. We therefore hypothesize:

**Hypothesis 1** **(H1).** *Witnessing online collective efficacy will correlate positively with enacting self-help upon encountering cyberhate*.

Formal social control of online content should also affect the likelihood of enacting self-help. Social networking sites determine what content is permissible on their platforms, and content can be flagged or removed, or users or groups suspended or deplatformed, for violating a site’s terms of service. Hence, website administrators have broad content control. Until recently, however, most social media sites have presented themselves as arenas of free speech, scantly regulating hateful speech on their platforms [51]. Even as social media titans, such as Facebook-cum-Meta and Twitter, step up efforts to remove hate speech from their sites, their attempts have largely been ineffectual. For one, what constitutes cyberhate is arguably subjective, and thus simply deciding what speech to regulate is challenging. Additionally, cyberhate is increasing [62,63], and the sheer volume of hateful material makes systematic monitoring untenable. Further complicating matters, detection algorithms are imperfect, vulnerable to the nuances of cyberhate, such as the use of sarcasm to attack or demean others. Relatedly, purveyors of hate are becoming adept at camouflaging their language to avoid discovery.

While noting the difficulties of content regulation, we expect witnessing website administrators to enact formal social control to affect the likelihood of users engaging in self-help. However, whereas we hypothesize a positive association between collective efficacy and self-help, we suspect that formal social control may reduce the likelihood of self-help. To understand why, we draw on Black’s [64] influential work, *Behavior of Law*, in which he argues that formal and informal social control are inversely related. Black [64] describes the relationship between self-help, or informal social control, and the law, or formal social control, as an ancient struggle [65]. Rooted in the Hobbesian [66] belief that lawlessness leads to chaos, Black [65] contends that self-help is more likely when formal social control is lax. Conversely, when formal social control is prevalent, individuals see less need to take the law into their own hands or engage in self-help. Additionally, the extant research on bystander intervention suggests bystander hesitancy when unsure of how best to intervene or when others intervene who they judge more qualified to offer assistance. It is likely that online users who see website administrators confronting cyberhate will not only deduce that the problem is being redressed, but also that it is being undertaken by a capable authority. We therefore hypothesize:

**Hypothesis 2** **(H2).** *Witnessing website administrators deleting or otherwise halting cyberhate will correlate negatively with enacting self-help upon encountering cyberhate*.

Finally, we controlled for two additional forms of informal social control—online and offline social bonds. Evaluating the potential effect of social bonds on enacting self-help is important because previous research has linked pro-social online behaviors to having a strong sense of community [15,25,48,67], and online and offline social bonds can provide friendship, support, and even protection [48]. Online, for instance, groups can serve as guardians, insulating members from cyberattacks, while also potentially emboldening group members to confront abusive online behavior. Likewise, attachment to primary groups offline, such as family and friends, can make online intervention more likely if users believe they have a strong support system to turn to if, for instance, they become targets of cyber-abuse. We therefore put forth the following two hypotheses:

**Hypothesis 3** **(H3).** *Closeness to an online community will correlate positively with enacting self-help upon encountering cyberhate*.

**Hypothesis 4** **(H4).** *Closeness to primary groups will correlate positively with enacting self-help upon encountering cyberhate*.

## 2. Materials and Methods

### 2.1. Sample and Data Collection

We analyzed a sample of 958 Internet users in the United States between the ages of 18–26 for this study. This age range was examined because young adults are avid Internet users and thus at an elevated risk of encountering online hate materials, all else being equal [68]. Data were collected in 2018 between 8 May and 18 May by Survey Sampling International (now Dynata). Dynata uses a number of permission-based recruiting techniques to find potential participants, including random digit dialing and banner ads. These online proportional sampling panels, such as the one used in this study, are pre-recruited and demographically balanced. They are found to have similar levels of reliability to probability sampling techniques specifically looking to assess attitudes [69,70].

The comprehensive survey, developed by the research group, asked respondents to respond to over 100 questions regarding their sociodemographic characteristics, online habits and routines, experiences with cyberhate and other anti-social online content, and various measures that estimate general personality traits and worldviews. Upon consenting, individuals were told they would be partaking in a study exploring the expression of extremist and hateful options online, and that the researchers are particularly interested in investigating individuals’ experiences with hateful or extremist content on social networking sites. Online proportional sampling panels were utilized to acquire data that were demographically balanced on important population characteristics. However, since the sample disproportionately represented females, we constructed sample weights based on the percentage of females in the United States between the ages of 18–26 in 2018. The weighted data were used in all analyses in this study.

### 2.2. Dependent Variable

The dependent variable measured pro-social online bystander action when encountering a hateful online attack. We argue that it is representative of the concept of self-help in response to seeing extremist content, in line with parallel work likewise conceptualizing self-help [48]. It is an average mean index constructed from two survey questions: (1) When people on social networking sites are being mean or offensive, how often do you tell the person who is being mean or offensive to stop?, and (2) When people on social networking sites are being mean or offensive, how often do you defend the person or group being attacked? Both questions have potential responses ranging from 1 (never) to 4 (frequently), with higher scores indicating a higher self-reported frequency of intervening during cyberhate attacks. To provide a sense of how the sample responded to the items included in this dependent variable indicator, seventeen percent of online survey-takers responded that they frequently tell online attackers to stop their actions, while nearly one-third said they never do so. Interestingly, respondents were slightly more likely to say they frequently defend the attacked, with nearly one-fifth registering a 4 for this question. Just under a quarter of respondents said they never act in defense of those being attacked by mean or offensive material online.

### 2.3. Independent Variables

Our key independent variables measured online and offline informal and formal social control. Online social control was assessed with two measures of online intervention. First, informal social control by others, collective efficacy, was measured as the average score of two items: one asked how frequently respondents see others defend the attacked person or group when people on social networking sites are being mean or offensive, and the other queried how frequently others tell the cyber-attacker to stop when people on social networking sites are being mean or offensive. Second, we controlled for formal social control with a measure of witnessing intervention on the part of website administrators. Respondents were asked how frequently they see site administrators delete offensive comments or otherwise halt mean or offensive online behavior. All three items had responses ranging from 1 (never) to 4 (frequently).

We controlled for two types of social bonds as indicators of social control. Offline social bonds were measured by asking respondents about their closeness to primary groups, while online social control was measured by asking about closeness to an online community. Closeness to primary groups was measured by taking the average of two indicators—closeness to family and closeness to friends. Closeness to an online community was assessed with a question asking survey-takers how close they feel to an online community to which they belong. Closeness to friends, family and an online community had response sets ranging from 1 (not at all close) to 5 (very close). Similar indicators have been used as measures of social control in numerous studies exploring various aspects of hateful online behavior [25,46,48].

Additionally, we controlled for several individual level factors that can influence enacting self-help, including sociodemographic and emotive traits as well as experiences with cyberhate. Four sociodemographic traits were included: age was measured in years, ranging from 18–26 years old, and sex (male = 1, female = 0), sexual orientation (heterosexual = 1, non-heterosexual = 0) and religious denomination (Christian = 1, non-Christian = 0) were binary variables. These measures could be important because past work on bystander intervention demonstrates that factors, such as sex, religious faith and age, are related to the likelihood of intervention [35,37,71]. Additionally, most cyberhate is presently associated with far-right extremism in the United States [72,73], which regularly targets women, non-Christians and members of the LGBTQIA+ community. Frequent targets of cyberhate could be more disturbed by its existence and more sympathetic to its victims, in turn, being more likely to engage in self-help.

An individual’s level of empathy was gauged with c composite measure created from five items. All five measures used the same response set, ranging from 1 (never) to 5 (always), and presented statements to the survey-takers who were asked to rate how frequently they felt or acted in the manner described. The statements are: (1) It upsets me to see someone being treated disrespectfully; (2) I enjoy making other people feel better; (3) I have tender, concerned feelings for people less fortunate than me; (4) I can tell when others are sad even when they do not say anything; and (5) I find that I am “in tune” with other people’s moods. The indicators were highly correlated with a McDonald’s omega coefficient of 0.82, and the factor analysis of the five dimension items revealed they load onto one factor with loadings between 0.7006 and 0.7659. We controlled for empathy with the expectation that individuals who demonstrate compassion towards others will be more likely to engage in self-help when witnessing a cyberhate attack. In fact, there is some evidence that empathy leads to bystander intervention under certain circumstances [44].

Experiences with cyberhate were captured two ways. First, respondents were asked how frequently they see material online that expresses negative views toward some group. Reponses ranged from 1 (never) to 4 (frequently). Second, respondents were asked how frequently they are personally attacked online due to any of nine traits that correspond to a cyberhate attack. These traits included ethnicity or race, nationality, sexual orientation, religious conviction/belief, political views, disability, sex or gender, gender identity and appearance. Survey-takers could check all that apply. Possible responses ranged from 1 (never) to 5 (11 times or more). Controlling for experiences with cyberhate is important because the extant work suggests personal encounters with negative online behaviors affect how individuals respond to such behaviors [25,74,75]. Frequent encounters with cyberhate can desensitize online users to its potential harm, as evidence suggests repeated exposure to violent media can numb its effects on viewers [76,77,78]. Further, there is the possibility that repeated exposure leads individuals to adopt views sympathetic to hate [6,9]. Hence, those who are frequently exposed to cyberhate should be less likely to engage in online self-help. An alternative is possible, though; that is, online users who are frequently targeted by cyberhate may be more cognizant of its harmful effects, and thus more likely to intervene to aid victims. In fact, a recent study found online users who experience cyberhate attacks are more disturbed by cyberhate generally [25].

Lastly, we controlled for basic online habits to assess whether the likelihood of intervention is partially a function of how individuals utilize the Internet. First, we measured the number of hours per day survey respondents spend on the Internet using an ordinal indicator. Possible responses ranged from 1, “less than one hour per day”, to 6, “ten or more hours per day”. Second, social network usage was measured by asking respondents which sites they utilized in the three months prior to being surveyed. Respondents were provided the option to choose all that apply from a comprehensive list of twenty-two social networking sites (We tested several versions of this variable, including using a dichotomized version with social network site usage coded as high or low. The results remained consistent regardless of how social networking usage was measured). Controlling for these measures is important because Internet habits have been linked to experiences with hateful online content [46,79]. This could affect how and if individuals respond to seeing others being targeted by cyberhate attacks, although we do not propose specific expectations regarding online habits and engaging in self-help.

Table 1 reports the means, standard deviations and minimum and maximum values for all variables in the analysis. A correlation matrix (available upon request) was used to initially assess possible sources of multicollinearity. We used a correlation above 0.6 as a source of concern; all correlations were below this threshold. We additionally conducted a variance inflation factor (VIF) test, which confirmed a lack of multicollinearity (mean VIF score = 1.17, with all individual VIF scores below 2).

### 2.4. Analytic Strategy

We used an ordinal logistic regression technique to analyze factors associated with engaging in self-help upon encountering cyberhate. This technique was appropriate because our dependent variable was categorical, measured at the ordinal-level, using a 4-point Likert scale to gauge frequency of engaging in online self-help. The effects of independent variables were reported as both regression coefficients and odds ratios. Odds ratios show relative changes in the odds of an outcome when an independent variable’s value is increased by one unit, holding all other effects constant. We included odds ratios because they are more easily interpretable when utilizing logistic regression. We conducted our analysis in a two-model sequence. The first model controlled for individual-level and target factors as well as online habits, and the second model added variables gauging online and offline social control. All analyses were conducted using STATA 15.1.

## 3. Results

Table 2 shows the results of regressing self-help when encountering cyberhate on the independent variables. The first model shows mixed support for our expectations. Individuals who identify as heterosexual are less likely to engage in self-help (OR = 0.69, *p* < 0.01), as expected, but in contrast to expectations, those who identify as Christian are more likely to do so (OR = 1.28, *p* < 0.05). Sex and age are not significantly related to the likelihood of engaging in online self-help. Thus, we do not find robust support for our expectation that individuals with sociodemographic traits readily targeted by cyberhate attacks will be more likely to engage in online self-help. As expected, those who score higher on our measure of empathy are more likely to engage in self-help (OR = 1.76, *p* < 0.001), suggesting empathetic individuals might be more attuned to the plight of those targeted by cyberhate attacks and therefore intervene.

Furthermore, those who see cyberhate frequently are more likely to engage in self-help (OR = 1.39, *p* < 0.001) as are those targeted by cyberhate more often (OR = 2.12, *p* < 0.001). In fact, those who are readily targeted are more than twice as likely to engage in self-help, relative to those who have not been targeted by cyberhate or are targeted with less regularity. These findings align with the expectation that those who come into contact with cyberhate more often may feel sympathy towards others who are targeted or may believe cyberhate is a persistent problem in need of intervention. Spending more time online and social network site usage are not significantly related to our outcome of interest at traditional levels of significance (Hours online is negatively related to self-help at the 0.10 level, while social network usage is positively related to self-help at the 0.10 level).

The second model demonstrates general support for our expectations regarding social control and online self-help. Strong ties to primary groups (OR = 1.20, *p* < 0.01) and online communities (OR = 1.17, *p* < 0.001) are both associated with engaging in online self-help. These findings suggest that individuals with a strong support system might feel emboldened to act in pursuit of assisting others. Likewise, those who witness informal social control in the form of collective efficacy are nearly three times as likely to enact self-help (OR = 2.83, *p* < 0.001), though witnessing site administrators delete cyberhate content is not significantly related to enacting self-help. Most of the results from the prior model remain consistent in this model. The lone change is that hours online become significant at traditional levels of significance (OR = 0.90, *p* < 0.05).

We examined the post-estimation statistics of our ordinal logistic regression models, including Akaike Information Criterion (AIC) and Bayesian Information Criterion (BIC), as well as Nagelkerke’s R^2^. Both AIC and BIC figures were smaller in Model 2 compared to Model 1, suggesting the fuller model is a better fit. Likewise, Nagelkerke’s R^2^ increased from Model 1 to Model 2, demonstrating that the second model explains more variation regarding engaging in self-help online.

## 4. Discussion

The main objective of this study was to assess the role of pro-social behavior in response to observing cyberhate. Understanding when and if individuals engage in self-help is important considering the increasing amount of cyberhate and the expected growth in time that youths will spend online in the coming decades. The analysis confirmed most of our core predictions. First, witnessing others enact social control apparently encourages others to intervene, supporting Hypothesis 1. As has been found in other settings, victims with allies are more likely to be supported and defended [33]. Indeed, those witnessing online collective efficacy were substantially more likely to defend someone being attacked when they witnessed this behavior online. This suggests that collective efficacy is built from the ground up and through equal peers, which makes it contagious. Just as offline communities with high levels of collective efficacy enjoy low crime rates and other social benefits in part because members trust each other to act on the community’s behalf, members of online communities where the community defends others fosters an environment in which people are willing to contribute to the common good.

The findings that being close to a primary group and an online community correlate with self-help support Hypotheses 3 and 4, and further buttress the notion that those with allies are more likely to act. In both cases, being embedded in a support network affords one the freedom to act. This may be because intervening in an online attack in defense of a victim can be dangerous. By standing up for others, one increases their target gratifiability and therefore their likelihood of victimization [48] by possibly irritating the offender. Hence, defending others can turn the wrath of the offender toward the defender. This can be a precarious situation if one is alone; however, when one has allies, they are likely more confident those allies will defend them should the need arise. In short, there is strength and security in numbers, another benefit of collective efficacy. Yet, not only are those with allies likely to be defended should the aggressor turn his or her attention toward them, they may very well be encouraged to or rewarded for their efforts to support a victim. Again, this demonstrates that pro-social behavior is contagious.

Next, we find modest support for Black’s [64] claim that informal social control and formal social control are inversely related. We anticipated a negative correlation between witnessing formal social control and self-help; the relationship was non-significant, however, failing to support Hypothesis 2. The fact that witnessing a site administrator intervention is unrelated to enacting self-help suggests that people are not compelled to assist a victim when an authority figure is present. It may also speak to the ability of online users to morally disengage from instances of cyberhate targeting others. Indeed, several mechanisms of moral disengagement may be relevant in this case [80]. For instance, some online users may not feel compelled to intervene, even viewing forms of anti-social behavior as important because they reaffirm the “right” to speak freely online. Additionally, online users who witness cyberhate could make an advantageous comparison, reasoning that some forms of cyberhate are not as harmful as other types of injurious online behavior, such as cybercrime, and therefore unworthy of intervention. Dehumanization might also play a role. Cyberhate is generally dehumanizing, targeting victims in the aggregate, and deeming them worthy of hate based on a particular group-level trait. Thus, online users who agree with the cyberhate they witness will be less likely to offer assistance via self-help. Finally, since the Internet is largely anonymous, it is easier to either displace or diffuse responsibility for helping others. Of note, if someone reasons that an authority figure is adequately addressing a problem, or that another online user is more likely to do so, and will likely do so more effectively, they may conclude their intervention is not needed or could even be detrimental.

Inaction on the part of users could allow cyberhate to spread, though. While popular social media sites, such as Facebook-cum-Meta, Twitter and YouTube, have, at times, made efforts in earnest to diminish hateful content on their platforms, their efficacy remains an issue of debate. Social media content is notoriously difficult to regulate; editorial oversight is generally absent, and the speed and volume of content creation make detection daunting. Complicating matters, purveyors of hate are often savvy, finding ways to evade hate-detection algorithms through the use of coded and ever-changing language, or by embedding hate in pictures or memes, staying one step ahead of detection mechanisms. Thus, it is not clear if witnessing a successful act of administrator intervention is indicative of the broader ability of formal social media policing. In sum, relying solely on site administrators to regulate hate speech is likely an ineffectual strategy. Online users undoubtedly play a crucial role in effectively policing online spaces.

Additionally, we find that experiences with cyberhate affect a user’s likelihood of enacting self-help. This is even more important since there is scant evidence of existing effective interventions to tackle cyberhate [25,81]. Specifically, seeing and being targeted by cyberhate frequently correlates with pro-social interventions. Seeing cyberhate regularly may relate to bystander intervention because it leads to the belief that online hate is a pervasive problem. Thus, the impetus to remedy the situation may be increased. It is plausible that those who rarely see cyberhate recognize it as a minor concern, or no concern at all, and do not seek avenues of redress. Being targeted frequently by cyberhate also may relate to bystander intervention because online users who endure attacks likely recognize their potential harm. Similarly, this might help us to understand why members of the LGBTQIA+ community, regular targets of cyberhate [25], are likewise more apt to intervene upon encountering hate. Interestingly, these results are significant even after controlling for empathic personality traits, which also positively correlate with bystander intervention. Hence, it is not solely compassion for others that drives frequent witnesses or targets of cyberhate to assist those under attack.

### Study Limitations

We believe this study has many strengths; yet, it is not without limitations. One potential limitation pertains to the sample. While the age range of study participants, 18–26, limits the generalizability of our findings, it also builds upon prior work on cyber-abuse that focuses disproportionately on adolescents. Moreover, young adults spend an inordinate amount of time online, and therefore the potential to be exposed to and respond to cyberhate is great among this demographic.

Second, we utilized demographically balanced panel data, allowing for a representative demographics of U.S. citizens. It is possible, however, that panel participants may have characteristics that differentiate them from individuals who chose not to participate. While this is a limitation of most survey-based research and we believe our sample is representative of theoretically important groups, we cannot determine if other biases related to this sampling procedure are present. We are nevertheless confident, given the frequent use of panel data for studies such as ours, that our results are valid and important. Additionally, expanding the scope to see if these patterns are replicated in other samples, such as in other countries, would help to further understand any bias in the sample.

Third, several of our measures were based on the subjective interpretation of our respondents since they were self-reported. For instance, we asked individuals to determine if they witnessed someone else being victimized by hate material online, and hate material can be perceived differently. This concern is not unique to this study, of course, as survey-based research often relies on the subjective interpretation of complex ideas by study participants. Additionally, a preliminary investigation into how online users understand the definition of cyberhate suggests that users do in fact generally agree on a definition of hate [68], which indicates that the measures utilized in this study have some reliability.

## 5. Conclusions

Our work demonstrated that pro-social online behavior is contagious. This finding, perhaps at present more than ever, is important. Indeed, when tech-titan Mark Zuckerberg announced in 2021 that Facebook would be rebranded *Meta* and subsequently launch a *metaverse*, he was foretelling the next step in the evolution of digitized existence [82]. As Meta and other social media platforms become increasingly ubiquitous in our lives, we should expect—for better and for worse—individuals to spend more time online. A predictable adverse consequence is heightened exposure to unwanted and harmful content, including hate material. It is therefore critical to accelerate efforts to understand both what fosters anti-social online behavior and also when and how to effectively respond to it. This work suggests that bystander intervention can have positive outcomes through fostering virtual communities that do not condone cyberhate. When online users speak up on behalf of cyber-victims, others become more likely to act accordingly. Of course, online bystander intervention is not without risks; interveners can become targets, or feed online trolls who are engaging in cyberhate purely for amusement. Yet, it seems the power of collective efficacy is stronger than the costs of intervening. To allow cyberhate to spread online unabated can serve to normalize it and accelerate attacks against members of vulnerable groups.

## Figures and Tables

**Table 1:** Descriptive statistics of all variables.

Variable	Mean or %	Std. Dev.	Min. Value	Max. Value
Self-Help	2.35	0.96	1	4
Male = 1	51%	0.50	0	1
Age	21.92	2.22	18	26
Heterosexual = 1	67%	0.47	0	1
Christian = 1	52%	0.50	0	1
Empathy	−0.07	1.01	−3.47	1.50
Frequency of Seeing Cyberhate	2.73	0.91	1	4
Frequency of Being Targeted by Cyberhate	1.73	0.88	1	5
Hours/Day Online	3.92	1.44	1	6
Social Network Site Usage	6.40	3.55	0	22
Closeness to Primary Group	4.01	0.94	1	5
Closeness to Online Community	2.84	1.26	1	5
Online Collective Efficacy	2.65	0.91	1	4
Online Formal Social Control	2.34	0.98	1	4

**Table 2:** Ordinal logistic regression analysis of enacting online self-Help.

	Model 1		Model 2	
Self-Help	Coef. (OR)	Std. Err.	Coef. (OR)	Std. Err.
Male = 1	0.08 (1.09)	0.14	0.13 (1.14)	0.15
Age	−0.02 (0.98)	0.03	−0.02 (0.98)	0.03
Heterosexual = 1	−0.37 (0.69) **	0.09	−0.39 (0.68) **	0.09
Christian = 1	0.25 (1.28) *	0.16	0.21 (1.24)	0.16
Empathy	0.57 (1.76) ***	0.13	0.30 (1.35) ***	0.11
Frequency of Seeing Cyberhate	0.33 (1.39) ***	0.12	0.20 (1.22) *	0.11
Frequency of Being Targeted by Cyberhate	0.76 (2.13) ***	0.17	0.65 (1.91) ***	0.18
Hours/Day Online	−0.08 (0.92)	0.04	0.10 (0.90) *	0.04
Social Network Site Usage	0.04 (1.04)	0.02	0.02 (1.02)	0.02
Closeness to Primary Group		-	0.18 (1.20) **	0.09
Closeness to Online Community		-	0.15 (1.17) **	0.07
Online Collective Efficacy		-	1.04 (2.83) ***	0.27
Formal Online Social Control		-	0.07 (1.08)	0.08
Log Pseudolikelihood	−1673.36		−1565.087	
Wald X^2^	183.15		364.87	
AIC/BIC	3429/3502		3168/3260	
Nagelkerke R^2^	22.2		37.7	
N	958		958	

* *p* < 0.05. ** *p* < 0.01. *** *p* < 0.001 (two-tailed tests).

## Data Availability

Data will be made available by authors if reasonable requests are made.

## References

[1] Rebollo-Catalan A., Mayor-Buzon V. (2020). Adolescent bystanders witnessing cyber violence against women and girls: What they observe and how they respond. Violence Women.

[2] Backe E.L., Lilleston P., McCleary-Sills J. (2018). Networked individuals, gendered violence: A literature review of cyberviolence. Violence Gend..

[3] European Institute for Gender Equality (2017). Cyber Violence Against Women and Girls.

[4] Peterson J., Densley J. (2017). Cyber violence: What do we know and where do we go from here?. Aggress. Violent Behav..

[5] Anderson M. (2018). A Majority of Teens Have Experienced Some Form of Cyberbullying.

[6] Cowan G., Mettrick J. (2002). The effects of target variables and setting on perceptions of hate speech. J. Appl. Soc. Psychol..

[7] Federal Bureau of Investigation (2011). Domestic Terrorism: Focus on Militia Extremism. https://www.fbi.gov/news/stories/2011/september/militia_092211.

[8] Federal Bureau of Investigation (2011). Sovereign Citizens: A Growing Domestic Threat to Law Enforcement. https://leb.fbi.gov/2011/september/sovereigncitizens-a-growing-domesticthreatto-law-enforcement.

[9] Foxman A.H., Wolf C. (2013). Viral Hate: Containing Its Spread on the Internet.

[10] Perry B. (2000). “Button-down Terror”: The metamorphosis of the hate movement. Sociol. Focus.

[11] Tynes B. (2005). Children, adolescents and the culture of online hate. Handbook of Children, Culture and Violence.

[12] Tynes B., Reynolds L., Greenfield P.M. (2004). Adolescence, race, and ethnicity on the Internet: A comparison of discourse in monitored vs. unmonitored chat rooms. J. Appl. Dev. Psychol..

[13] Fischer P., Krueger J.I., Greitemeyer T., Vogrincic C., Kastenmüller A., Frey D., Heene M., Wicher M., Kainbacher M. (2011). The bystander-effect: A meta-analytic review on bystander intervention in dangerous and non-dangerous emergencies. Psychol. Bull..

[14] Latané B., Nida S. (1981). Ten years of research on group size and helping. Psychol. Bull..

[15] Banyard V.L. (2008). Measurement and correlates of prosocial bystander behavior: The case of interpersonal violence. Violence Vict..

[16] Banyard V.L., Plante E.G., Moynihan M.M. (2004). Bystander education: Bringing a broader community perspective to sexual violence prevention. J. Commun. Psychol..

[17] Berkowitz A.D. (2002). Fostering Men’s Responsibility for Preventing Sexual Assault.

[18] Darley J.M., Latané B. (1968). Bystander intervention in emergencies: Diffusion of responsibility. J. Personal. Soc. Psychol..

[19] Katz J. (1994). Mentors in Violence Prevention (MVP) Trainer’s Guide.

[20] Bastiaensens S., Vandebosch H., Poels K., Van Cleemput K., DeSmet A., De Bourdeaudhuij I. (2014). Cyberbullying on social network sites. An experimental study into bystanders’ behavioural intentions to help the victim or reinforce the bully. Comput. Hum. Behav..

[21] De Smet A., Veldeman C., Poels K., Bastiaensens S., Van Cleemput K., Vandebosch H., De Bourdeaudhuij I. (2014). Determinants of self-reported bystander behavior in cyberbullying incidents amongst adolescents. Cyberpsychol. Behav. Soc. Netw..

[22] Freis S.D., Gurung R.A. (2013). A Facebook analysis of helping behavior in online bullying. Psychol. Pop. Media Cult..

[23] Gahagan K., Vaterlaus J.M., Frost L.R. (2016). College student cyberbullying on social networking sites: Conceptualization, prevalence, and perceived bystander responsibility. Comput. Hum. Behav..

[24] Obermaier M., Fawzi N., Koch T. (2016). Bystanding or standing by? How the number of bystanders affects the intention to intervene in cyberbullying. New Media Soc..

[25] Costello M., Hawdon J., Bernatzky C., Mendes K. (2019). Social group identity and perceptions of online hate. Sociol. Inq..

[26] Black D. (1998). The Social Structure of Right and Wrong.

[27] Schwartz S.H., Gottlieb A. (1980). Bystander anonymity and reactions to emergencies. J. Personal. Soc. Psychol..

[28] Schwartz S.H., Gottlieb A. (1980). Participation in a bystander intervention experiment and subsequent everyday helping: Ethical considerations. J. Exp. Soc. Psychol..

[29] Berkowitz A.D. (2010). Fostering healthy norms to prevent violence and abuse: The social norms approach. The Prevention of Sexual Violence: A Practitioner’s Sourcebook.

[30] Karakashian L.M., Walter M.I., Christopher A.N., Lucas T. (2006). Fear of negative evaluation affects helping behavior: The bystander effect revisited. N. Am. J. Psychol..

[31] Latane B., Darley J.M. (1968). Group inhibition of bystander intervention in emergencies. J. Personal. Soc. Psychol..

[32] Latané B., Darley J.M. (1970). The Unresponsive Bystander: Why Doesn’t He Help?.

[33] Ireland L., Hawdon J., Huang B., Peguero A. (2020). Preconditions for guardianship interventions in cyberbullying: Incident interpretation, collective and automated efficacy, and relative popularity of bullies. Comput. Hum. Behav..

[34] Van Cleemput K., Vandebosch H., Pabian S. (2014). Personal characteristics and contextual factors that determine “helping”, “joining in”, and “doing nothing” when witnessing cyberbullying. Aggress. Behav..

[35] Eagly A.H., Crowley M. (1986). Gender and helping behavior: A meta-analytic review of the social psychological literature. Psychol. Bull..

[36] Weitzman A., Cowan S., Walsh K. (2020). Bystander interventions on behalf of sexual assault and intimate partner violence victims. J. Interpers. Violence.

[37] Hardy S.A., Carlo G. (2005). Religiosity and prosocial behaviours in adolescence: The mediating role of prosocial values. J. Moral Educ..

[38] Machackova H., Dedkova L., Sevcikova A., Cerna A. (2018). Bystanders’ supportive and passive responses to cyberaggression. J. Sch. Violence.

[39] Olenik-Shemesh D., Heiman T., Eden S. (2017). Bystanders’ behavior in cyberbullying episodes: Active and passive patterns in the context of personal–socio-emotional factors. J. Interpers. Violence.

[40] Brody N., Vangelisti A.L. (2016). Bystander intervention in cyberbullying. Commun. Monogr..

[41] Patterson L.J., Allan A., Cross D. (2016). Adolescent bystanders’ perspectives of aggression in the online versus school environments. J. Adolesc..

[42] Machackova H., Pfetsch J. (2016). Bystanders’ responses to offline bullying and cyberbullying: The role of empathy and normative beliefs about aggression. Scand. J. Psychol..

[43] Henson B., Fisher B.S., Reyns B.W. (2020). There is virtually no excuse: The frequency and predictors of college students’ bystander intervention behaviors directed at online victimization. Violence Women.

[44] Herry E., Gönültaş S., Mulvey K.L. (2021). Digital era bullying: An examination of adolescent judgments about bystander intervention online. J. Appl. Dev. Psychol..

[45] Kleinsasser A., Jouriles E.N., McDonald R., Rosenfield D. (2015). An online bystander intervention program for the prevention of sexual violence. Psychol. Violence.

[46] Räsänen P., Hawdon J., Holkeri E., Keipi T., Näsi M., Oksanen A. (2016). Targets of online hate: Examining determinants of victimization among young Finnish Facebook users. Violence Vict..

[47] Allen J.M., Norris G.H. (2011). Is Genocide Different-Dealing with Hate Speech in a Post-Genocide Society. J. Int. Law Int. Relat..

[48] Hawdon J., Oksanen A., Räsänen P. (2017). Exposure to online hate in four nations: A cross-national consideration. Deviant Behav..

[49] Hawdon J., Costello M. (2022). Confronting Online Extremism: Strategies, Promises, and Pitfalls. Right-Wing Extremism in Canada and the United States.

[50] Gerstenfeld P.B. (2017). Hate Crimes: Causes, Controls, and Controversies.

[51] Ozalp S., Williams M.L., Burnap P., Liu H., Mostafa M. (2020). Antisemitism on Twitter: Collective efficacy and the role of community organisations in challenging online hate speech. Soc. Media+ Soc..

[52] Dovidio J.F., Piliavin J.A., Gaertner S.L., Schroeder D.A., Clark R.D. (1991). The Arousal: Cost-Reward Model and the Process of Intervention: A Review of the Evidence.

[53] Dovidio J.F., Piliavin J.A., Schroeder D.A., Penner L.A. (2017). The Social Psychology of Prosocial Behavior.

[54] Thornberg R. (2010). Schoolchildren’s social representations on bullying causes. Psychol. Sch..

[55] DeRosier M.E., Kupersmidt J.B., Patterson C.J. (1994). Children’s academic and behavioral adjustment as a function of the chronicity and proximity of peer rejection. Child Dev..

[56] Sampson R.J. (2021). Crime and public safety: Insights from community-level perspectives on social capital. Soc. Cap. Poor Communities.

[57] Sampson R.J., Raudenbush S.W., Earls F. (1997). Neighborhoods and violent crime: A multilevel study of collective efficacy. Science.

[58] Mazerolle L., Wickes R., McBroom J. (2010). Community variations in violence: The role of social ties and collective efficacy in comparative context. J. Res. Crime Delinq..

[59] Sampson R.J., Raudenbush S.W. (1999). Systematic social observation of public spaces: A new look at disorder in urban neighborhoods. Am. J. Sociol..

[60] Sampson R.J., Wikström P.O. (2008). The social order of violence in Chicago and Stockholm neighborhoods: A comparative inquiry. Order, Conflict, and Violence.

[61] Voelpel S.C., Eckhoff R.A., Förster J. (2008). David against Goliath? Group size and bystander effects in virtual knowledge sharing. Hum. Relat..

[62] Beirich H., Buchanan S. (2018). 2017: The Year in Hate and Extremism.

[63] Costello M., Hawdon J. (2018). Who are the online extremists among us? Sociodemographic characteristics, social networking, and online experiences of those who produce online hate materials. Violence Gend..

[64] Black D. (1976). The Behavior of Law.

[65] Black D. (1983). Crime as social control. American Sociological Review.

[66] Hobbes T., Missner M. (2016). Thomas Hobbes: Leviathan (Longman Library of Primary Sources in Philosophy).

[67] Oksanen A., Hawdon J., Holkeri E., Näsi M., Räsänen P. (2014). Exposure to online hate among young social media users. Soul of Society: A Focus on the Lives of Children & Youth.

[68] Bernatzky C., Costello M., Hawdon J. (2022). Who Produces Online Hate?: An Examination of the Effects of Self-Control, Social Structure, & Social Learning. Am. J. Crim. Justice.

[69] Simmons A.D., Bobo L.D. (2015). Can non-full-probability internet surveys yield useful data? A comparison with full-probability face-to-face surveys in the domain of race and social inequality attitudes. Sociol. Methodol..

[70] Weinberg J.D., Freese J., McElhattan D. (2004). Comparing data characteristics and results of an online factorial survey between a population-based and a crowdsource-recruited sample. Sociol. Sci..

[71] George D., Carroll P., Kersnick R., Calderon K. (1998). Gender-related patterns of helping among friends. Psychol. Women Q..

[72] Costello M., Barrett-Fox R., Bernatzky C., Hawdon J., Mendes K. (2020). Predictors of viewing online extremism among America’s youth. Youth Soc..

[73] Potok M. (2015). The Year in Hate & Extremism, 2010. Intelligence Report, 141. https://www.splcenter.org/fighting-hate/intelligence-report/2015/year-hateand-extremism-0.

[74] Cowan G., Hodge C. (1996). Judgments of hate speech: The effects of target group, publicness, and behavioral responses of the target. J. Appl. Soc. Psychol..

[75] Cowan G., Heiple B., Marquez C., Khatchadourian D., McNevin M. (2005). Heterosexuals’ attitudes toward hate crimes and hate speech against gays and lesbians: Old-fashioned and modern heterosexism. J. Homosex..

[76] Anderson C.A., Suzuki K., Swing E.L., Groves C.L., Gentile D.A., Prot S., Lam C.P., Sakamoto A., Horiuchi Y., Krahé B. (2017). Media violence and other aggression risk factors in seven nations. Personal. Soc. Psychol. Bull..

[77] Bushman B.J., Anderson C.A. (2009). Comfortably numb: Desensitizing effects of violent media on helping others. Psychol. Sci..

[78] Saleem M., Prot S., Anderson C.A., Lemieux A.F. (2017). Exposure to Muslims in media and support for public policies harming Muslims. Commun. Res..

[79] Hawdon J., Oksanen A., Räsänen P. (2015). Online Extremism and Online Hate.

[80] Bandura A., Barbaranelli C., Caprara G.V., Pastorelli C. (1996). Mechanisms of moral disengagement in the exercise of moral agency. J. Personal. Soc. Psychol..

[81] Windisch S., Wiedlitzka S., Olaghere A. (2021). PROTOCOL: Online interventions for reducing hate speech and cyberhate: A systematic review. Campbell Syst. Rev..

[82] Roose K. (2021). The Metaverse Is Mark Zuckerberg’s Escape Hatch. The New York Times.

